# Dr. Alonzo Garcelon

**Published:** 1907-01

**Authors:** 


Dr. Alonzo Garcelon, of Lewiston, Me., one of the oldest physicians in the country, died at the home of his daughter in Medford, Mass., December 8, 1906, aged 93 years. He graduated in medicine from the medical college of Ohio, Cincinnati, in 1839. He had been a member of the American Medical Association since 1853, and was chairman of the board of trustees most of the time from 1883 to 1901, when he was elected vice-president of the association. He was governor of Maine in 1878, being the only democratic governor ever elected in that state. He was not only a prominent physician but a public spirited citizen and had a very wide circle of friends, being well known throughout the country.

